# Supplementary material for: A convergent evolutionary pathway attenuating cellulose production drives enhanced virulence of some bacteria
Source: Nat Commun. 2024 Feb 21;15:1441. doi: 10.1038/s41467-024-45176-4 (PMC10881479; doi:10.1038/s41467-024-45176-4)
Supplement: Supplementary file 1 — Supplementary Information [file 41467_2024_45176_MOESM1_ESM.pdf]

Supplementary Information for

A convergent evolutionary pathway attenuating cellulose production drives enhanced virulence in some bacteria

Nguyen Thi Khanh Nhu[1,2,3#], M Arifur Rahman[3,4#a], Kelvin G. K. Goh[5,6#], Seung Jae Kim[7,8#], Minh-Duy Phan[1,2,3], Kate M. Peters[1,2,3], Laura Alvarez-Fraga[2,3b], Steven J. Hancock[2,3c], Chitra Ravi[1,2,3], Timothy J. Kidd[2,3d], Matthew J. Sullivan[5,6e], Katharine M. Irvine[3,4], Scott A. Beatson[2,3], Matthew J. Sweet[1,3], Adam D. Irwin[3,9,10], Jana Vukovic[7,8]*, Glen C. Ulett[5,6]*, Sumaira Z. Hasnain[3,4]*, and Mark A. Schembri[1,2,3]*

[1]Institute for Molecular Bioscience (IMB), The University of Queensland, Brisbane, Queensland, Australia

[2]School of Chemistry and Molecular Biosciences, The University of Queensland, Brisbane, Queensland, Australia

[3]Australian Infectious Diseases Research Centre, The University of Queensland, Brisbane, Queensland, Australia

[4]Immunopathology Group, Mater Research Institute, The University of Queensland, Translational Research Institute, Brisbane, Australia.

[5]School of Pharmacy and Medical Sciences, Griffith University, Southport, Queensland, Australia

[6]Menzies Health Institute Queensland, Griffith University, Southport, Queensland, Australia

[7]School of Biomedical Sciences, Faculty of Medicine, The University of Queensland, Brisbane, Queensland, Australia

[8]Queensland Brain Institute, The University of Queensland, Brisbane, Queensland, Australia

[9]University of Queensland Centre for Clinical Research, Brisbane, Australia

[10]Queensland Children's Hospital, Brisbane, Australia

# Contributed equally

[a]Current Address: QIMR Berghofer Medical Research Institute, Brisbane, Queensland, Australia.
[b]Current Address: INRAE, Univ Montpellier, LBE, 102 Avenue des Etangs, Narbonne 11100, France.
[c]Current Address: Wellcome-Wolfson Institute for Experimental Medicine, School of Medicine, Dentistry and Biomedical Sciences, Queen's University Belfast, Belfast, United Kingdom.
[d]Current Address: Central Microbiology, Pathology Queensland, Royal Brisbane and Women's Hospital, Brisbane, Australia.
[e]Current Address: School of Biological Sciences, University of East Anglia, Norwich, NR4 7TJ, UK.

***Corresponding authors**

Mark Schembri: Institute for Molecular Bioscience (IMB), The University of Queensland, Brisbane, Queensland 4072, Australia. Telephone: +61733653306; E-mail: m.schembri@uq.edu.au

Sumaira Z. Hasnain: Immunopathology Group, Mater Research Institute, The University of Queensland, Translational Research Institute, Brisbane, Australia. Telephone: +61734436939; E-mail: sumaira.hasnain@mater.uq.edu.au

Glen Ulett: School of Pharmacy and Medical Sciences, and Menzies Health Institute Queensland, Griffith University, Gold Coast, QLD 4222, Australia. Telephone: +61756780765; E-mail: g.ulett@griffith.edu.au

Jana Vukovic: School of Biomedical Sciences, Faculty of Medicine, The University of Queensland, Brisbane, Queensland, Australia. Telephone: + 61733652818; E-mail: j.vukovic@uq.edu.au

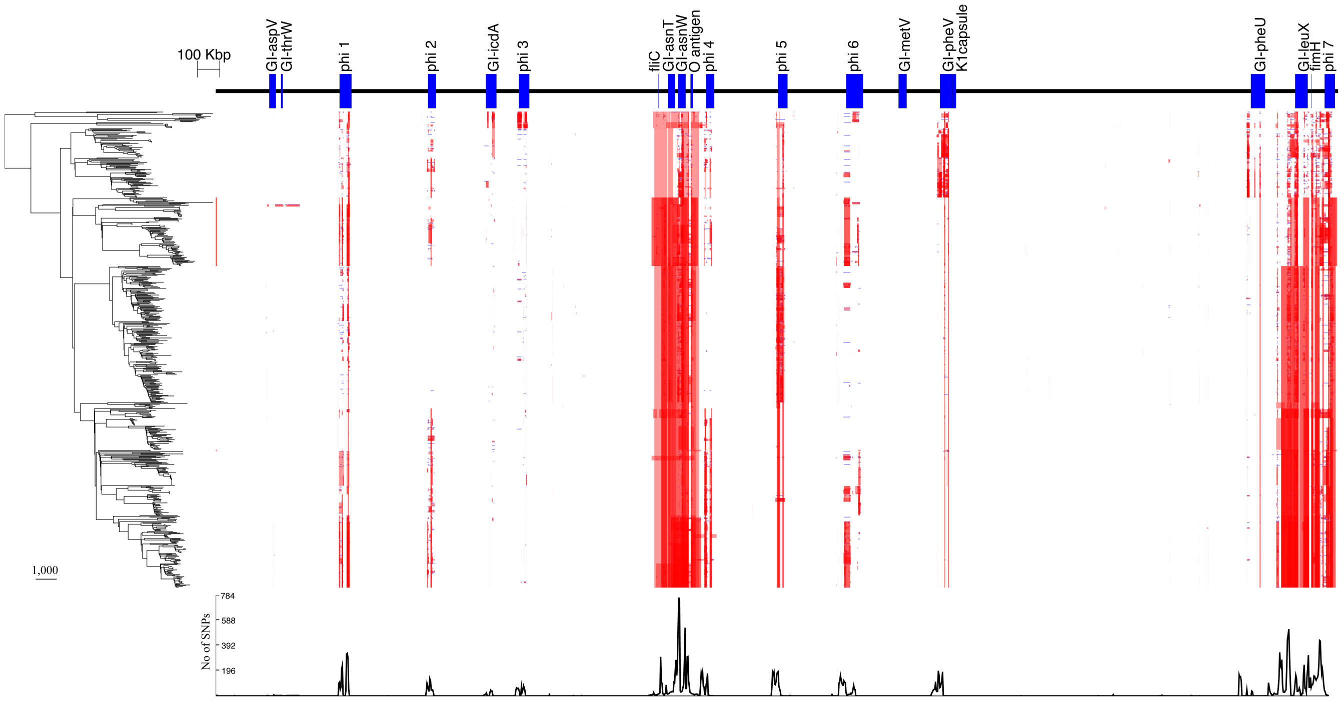

Supplementary Fig 1. Recombination regions in ST95. Gubbins analysis of ST95 whole genome alignment built by integrating SNPs in each strain to the MS7163 chromosome backbone (red, recombination detected in several isolates; blue, recombination specific to its taxa. Left panel represents the phylogenetic tree of ST95, top panel is MS7163 chromosome annotated with GIs, prophages and regions of interest. The middle section shows the number of SNPs according to the MS7163 chromosome backbone. Source data are provided as a Source Data file.

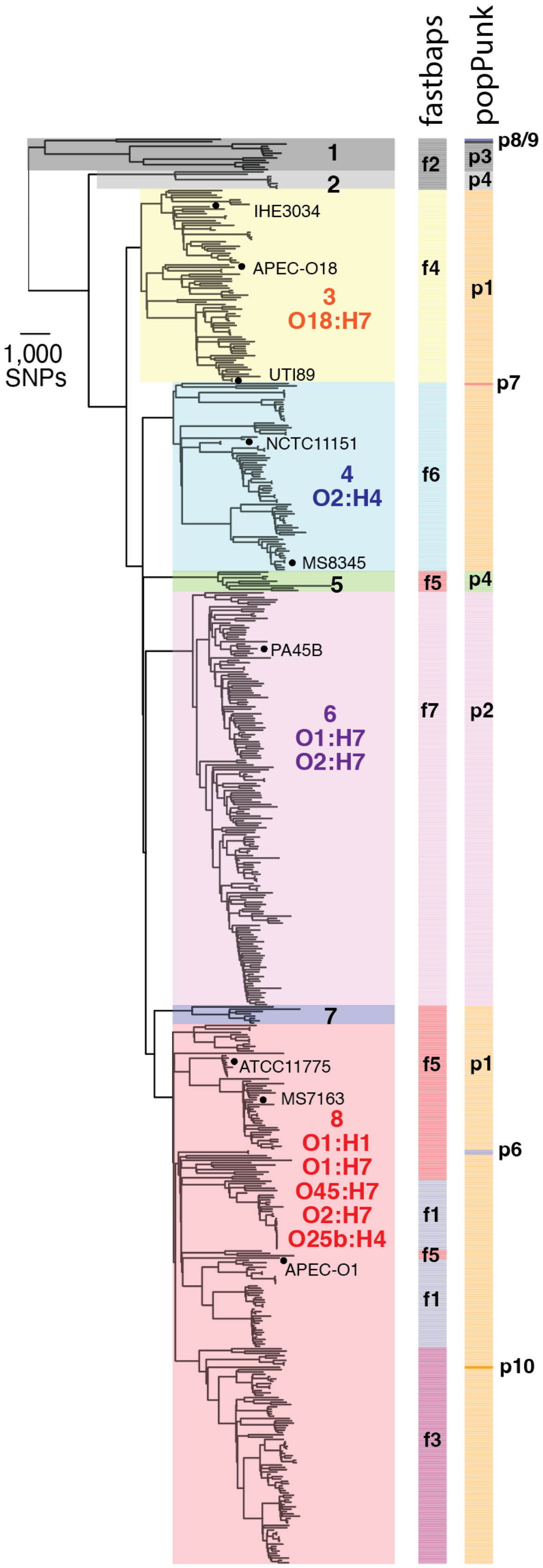

Supplementary Fig 2. ST95 clade designation by SNP phylogeny and population structure analysis by fastbaps and popPunk.

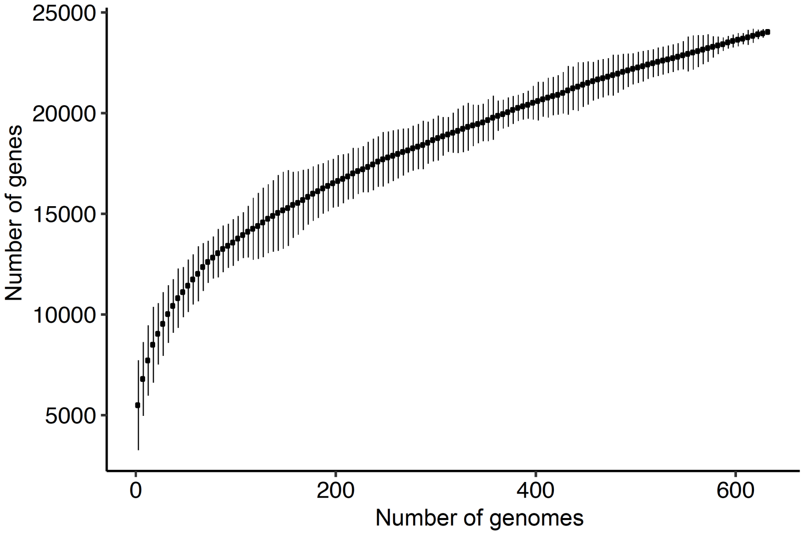

Supplementary Fig 3. ST95 pangenome accumulation plot. Plot of point range (mean +/- min, max, binwidth = 5) of number of genes in pan genome.

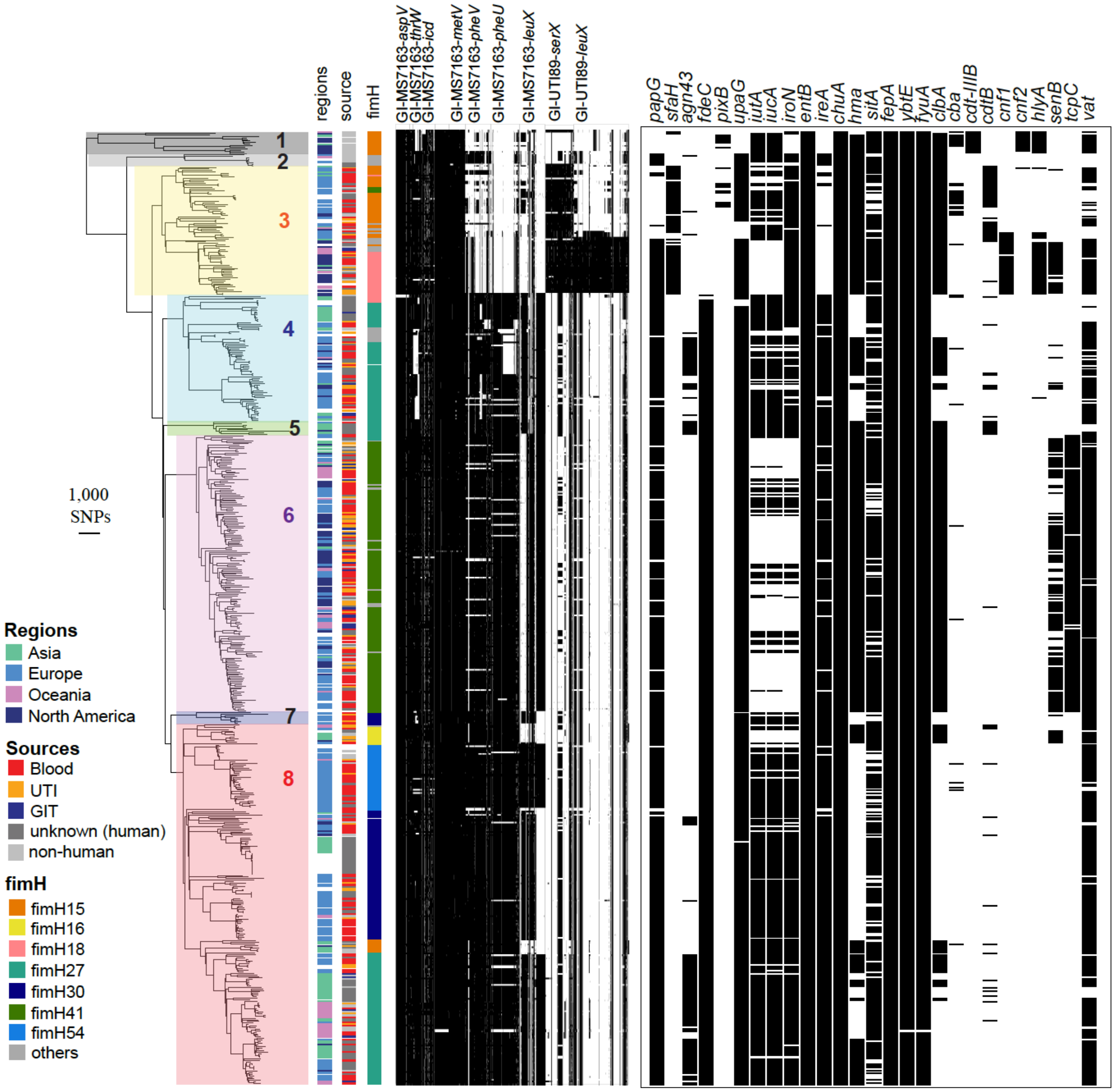

Supplementary Fig 4. Phylogeny of ST95 contextualized with respect to geographic origin, source, genomic islands and virulence factors. The isolation region and source of ST95 strains are color-coded according to keys (UTI, urinary tract infection; GIT, gastrointestinal tract infection). Conservation of GIs from MS7163 and UTI89 in ST95 is shown, with black lines indicating a match of >90% nucleotide conservation based on a blast search of ST95 assemblies against the reference with a 200-bp window (generated using seqfindr). The prevalence of selected *E. coli* virulence genes is also shown, with black lines indicating gene presence with > 90% nucleotide identity and > 80% gene coverage.

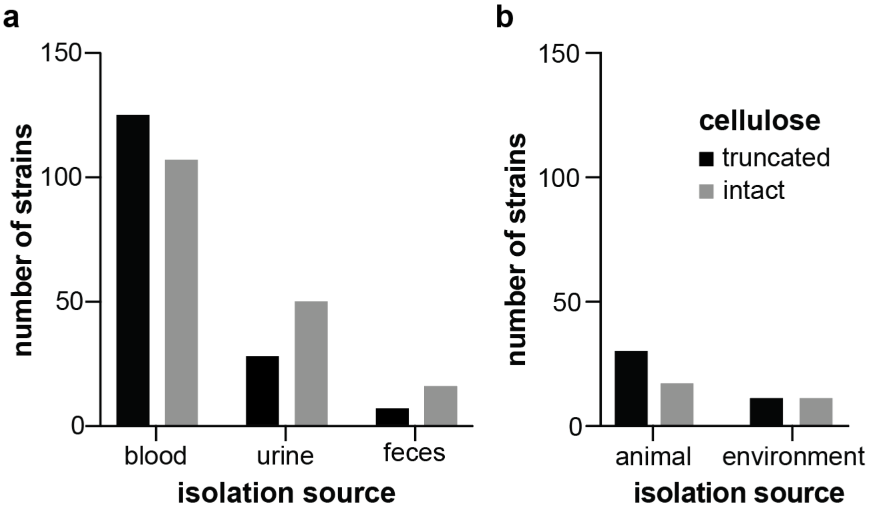

Supplementary Fig 5. Cellulose disruption is significantly associated with ST95 blood isolates compared to urine and fecal isolates. a, Number of ST95 isolates in our dataset that contain mutations in cellulose biosynthesis or modification based on their site of isolation (blood, urine and feces). In total, there were 333 human isolates for which there was appropriate metadata, comprising 232 blood, 78 urine and 23 fecal isolates. Statistical analysis revealed significant association between cellulose disruption in blood isolates compared to urine and fecal isolates (Chi-square test, 2 tailed P-value = 0.0049). **b,** Number of ST95 isolates originating from animals or the environment in our dataset that contain mutations in cellulose biosynthesis or modification. In total, there were 47 animal isolates and 22 environmental isolates. No significant associations were identified (Chi-square test, 2 tailed P-value = 0.3). Source data are provided as a Source Data file.

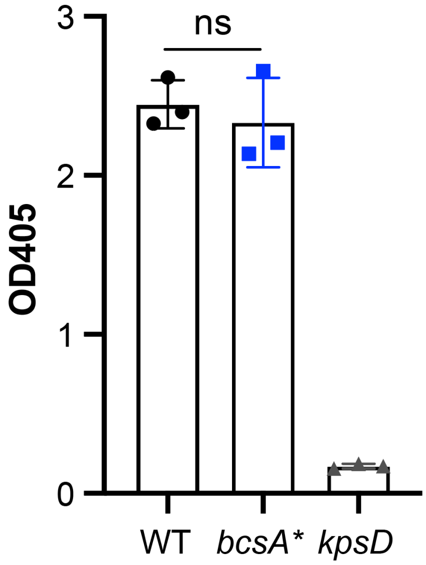

Supplementary Fig 6. WT and *bcsA** produce the same amount of K1 capsule. K1 capsule production was determined by ELISA using a monoclonal antibody specific for K1 ELISA. Data was shown as mean ± SD of three biological replicates. Source data are provided as a Source Data file.

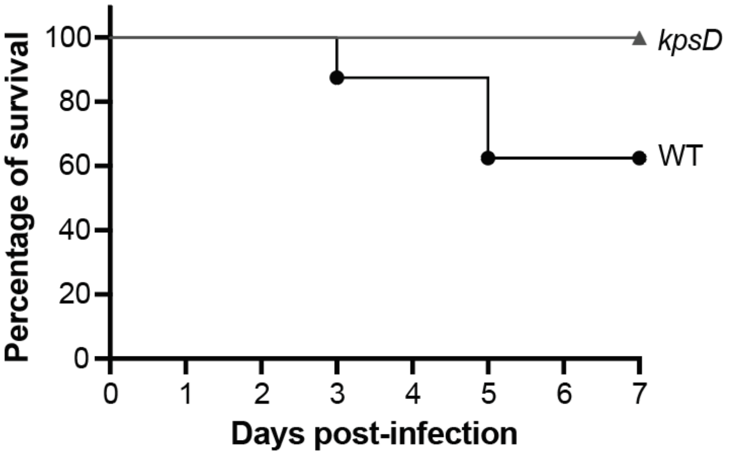

Supplementary Fig 7. Percentage of survival of pups during the 7-day infection period following infection at day 2 post birth (P2). Rat pups were infected with WT MS7163 (8 pups) or MS7163*kps*D (a K1 capsule mutant, 7 pups). Source data are provided as a Source Data file.

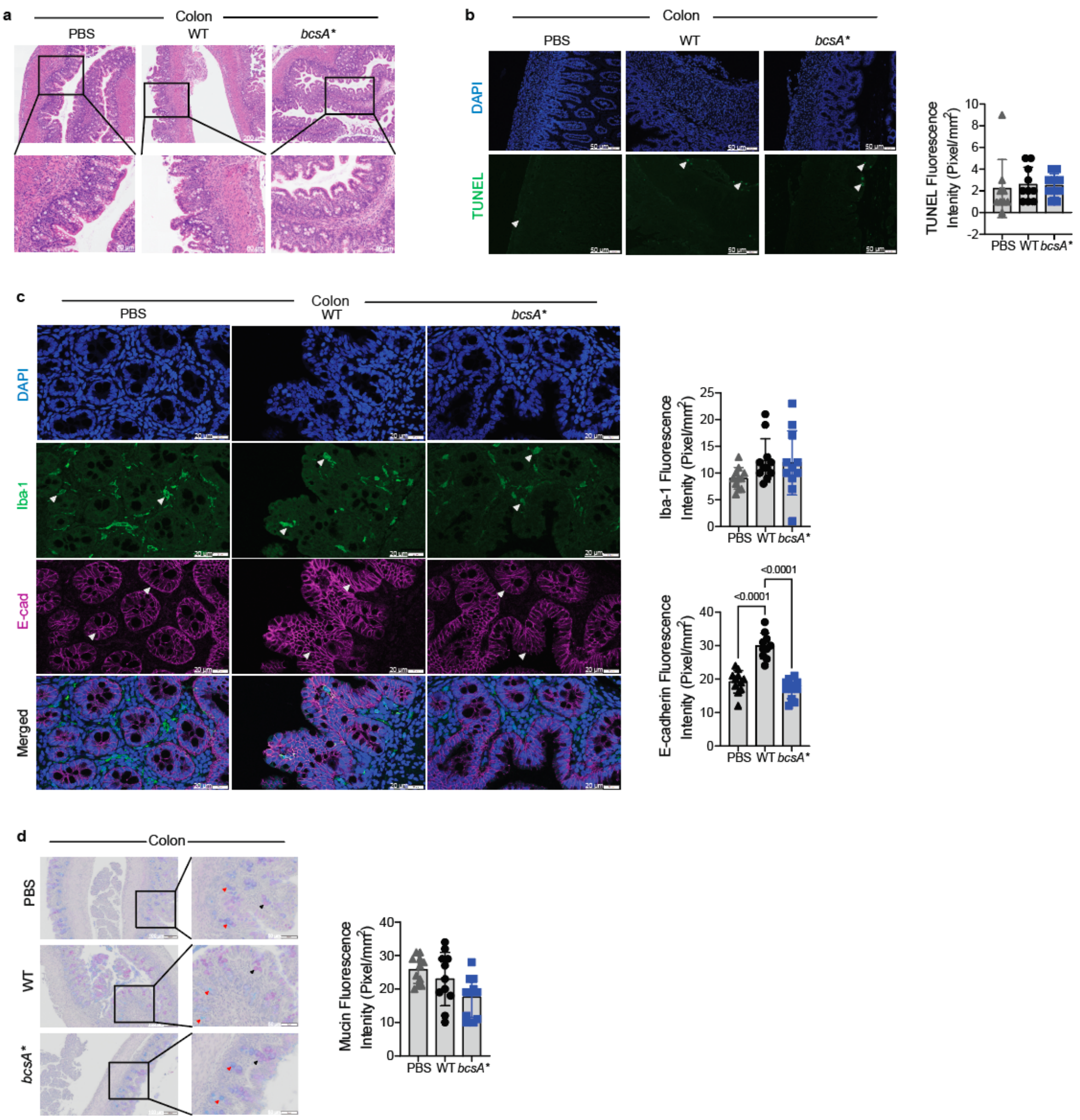

Supplementary Fig 8. Cellulose disruption enhances infection and inflammation in the neonatal rat colon. a, Representative tissue pathology (H&E) micrographs of mid colon (scale bars-200 µm in top panel and 80 µm in bottom panel). **b,** Apoptosis (TUNEL) staining images (white arrowheads indicating cell death, scale bars- 50 µm) and relative quantification of TUNEL fluorescence intensity (Pixel/mm^2) in the colon. **c,** Immune-fluorescent staining images showing macrophage (white arrowheads) infiltration and tight junction proteins, E-cadherin expression (white arrowheads) in the colon. **d,** Mucus secreting goblet cells (red arrowhead) and immature goblet cells (black arrowhead) by Alcian Blue-Periodic Acid Schiffs staining and relative Mucin fluorescence intensity quantification of the colon of 7-days old rat pups infected with PBS, WT and *bcsA**. Data are presented as mean ± SD (one-way ANOVA, two-three independent experiments, n=4-10). Source data are provided as a Source Data file.

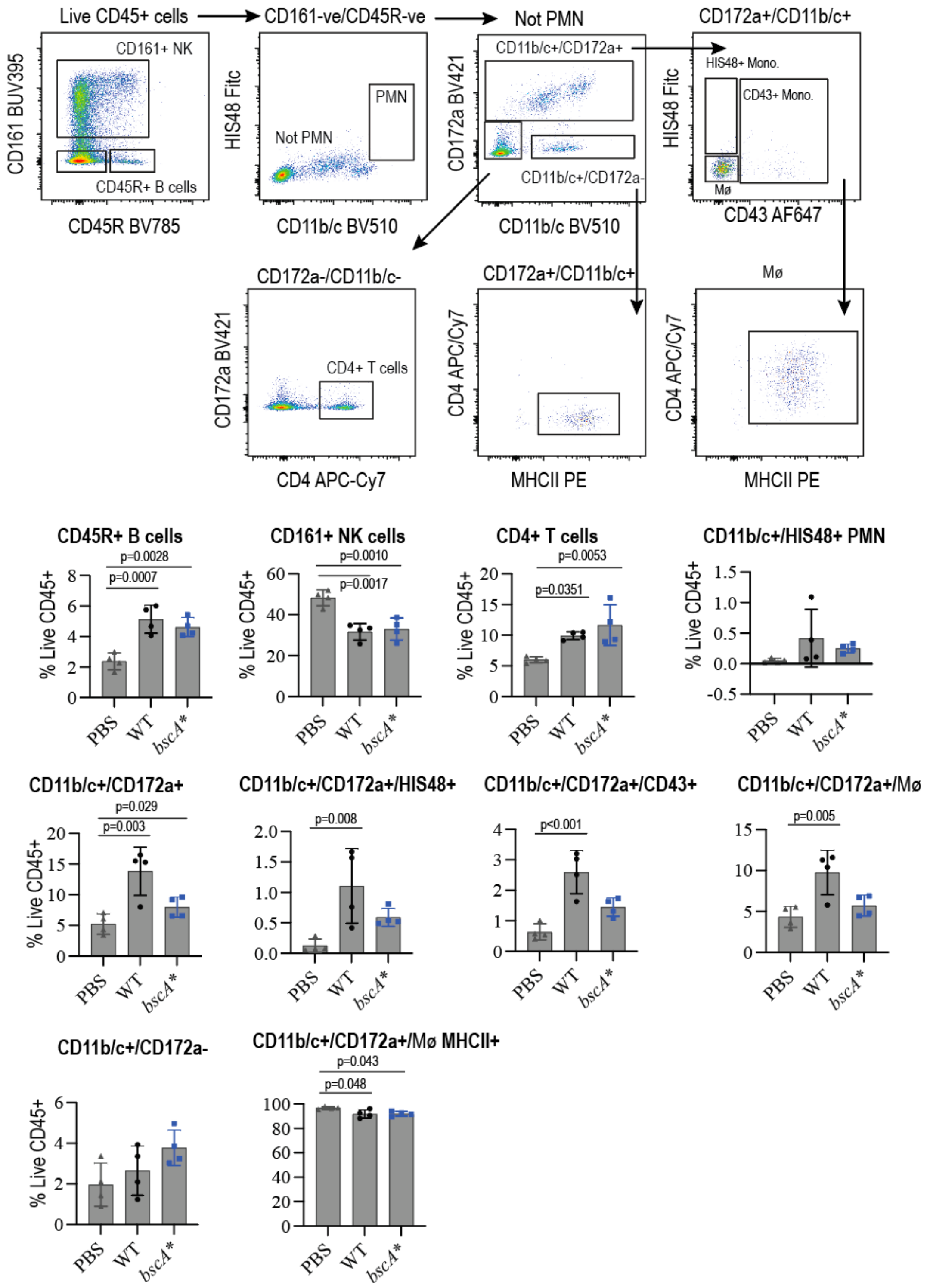

Supplementary Fig 9. Immune response in the neonatal rat colon. Flow cytometry gating strategy for identification and phenotyping of colon leukocytes, representative dot plots. NK cells were defined as CD161+, B cells as CD45R+ (B220) and neutrophils (PMN) as CD11b/cHi/CD172aLow (HIS48+). CD172aHi myeloid cells were gated to distinguish macrophages (CD4+ cells lacking monocyte markers), HIS48+ classical monocytes and CD43+ non-classical monocytes. CD4+ T cells were gated as a subset of non-NK, B, PMN and myeloid cells. **d,** Relative frequency of colon NK, B, T, polymorphonuclear (PMN) cells; monocyte and macrophage subsets and macrophage MHCII expression. Data are presented as mean ± SD (n=4). P-value are only shown for significant difference, determined by One-way ANOVA with Tukey's multiple comparison test. Source data are provided as a Source Data file.

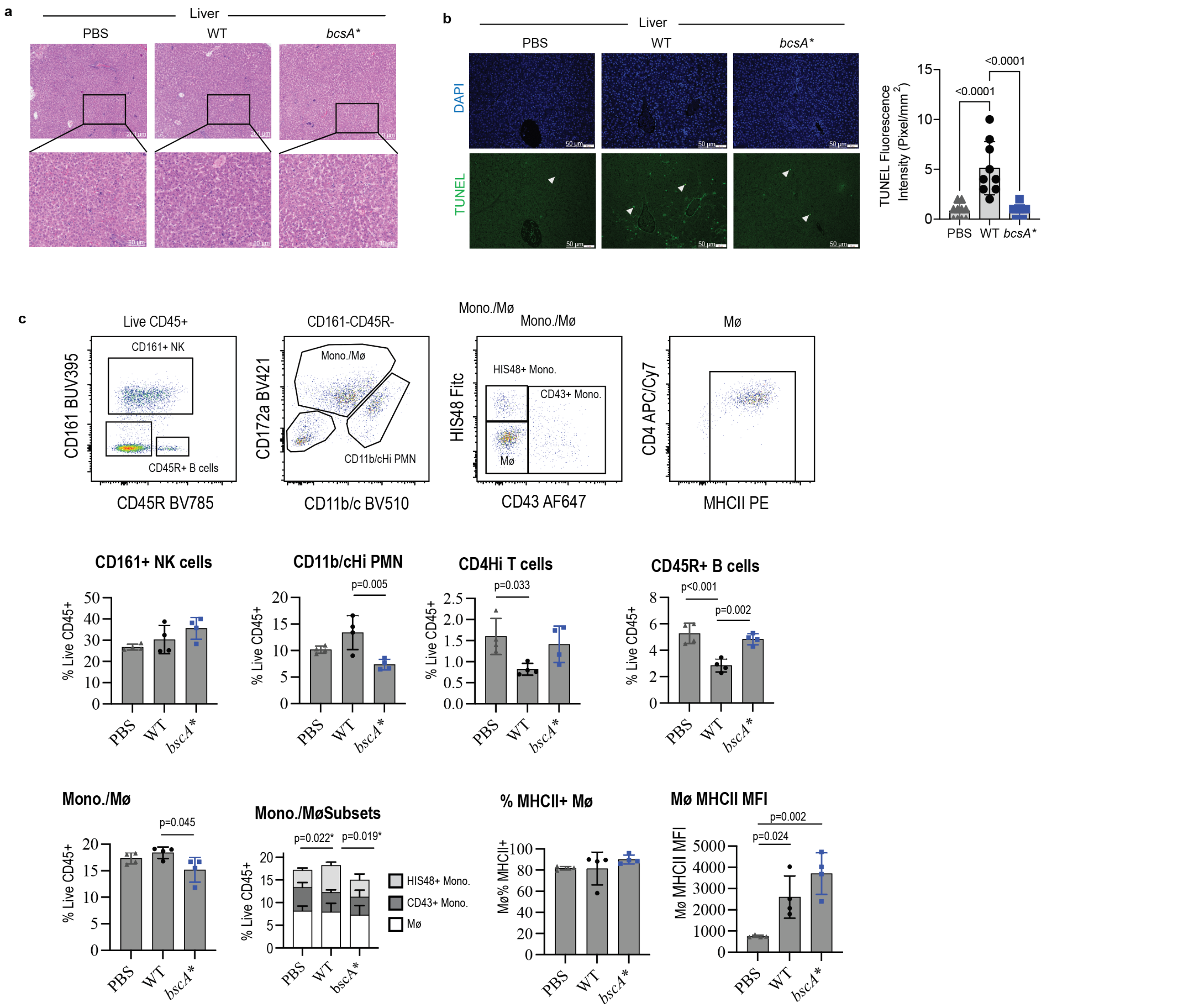

Supplementary Fig 10. Cellulose disruption induces hepatic inflammation and cell death. a, Representative tissue pathology (H&E) micrographs of the liver. **b,** Apoptosis (TUNEL) staining images and quantification showing relative cell death in the liver (n=4-10). **c,** Flow cytometry gating strategy for identification and phenotyping of liver leukocytes, representative dot plots (n=4). NK cells were defined as CD161+ and B cells as CD45R+ (B220). Few neutrophils (PMN), gated as CD11b/cHi/CD172aLow/HIS48+, were identified. The remaining myeloid cells fell into CD172a+/CD11b/c+ monocytes (HIS48+ classical and CD43+ non-classical) and macrophages (CD43-/HIS48-/CD4+/MHCII+) and a population of CD11b/c+/CD172a- cells, the majority of which were MHCII+. Relative frequency of liver NK, B, T, polymorphonuclear (PMN) cells; monocyte and macrophage subsets and macrophage MHCII expression. Data are presented as mean ± SD. P-values were calculated with one-way ANOVA with Tukey’s multiple comparison test. Source data are provided as a Source Data file.

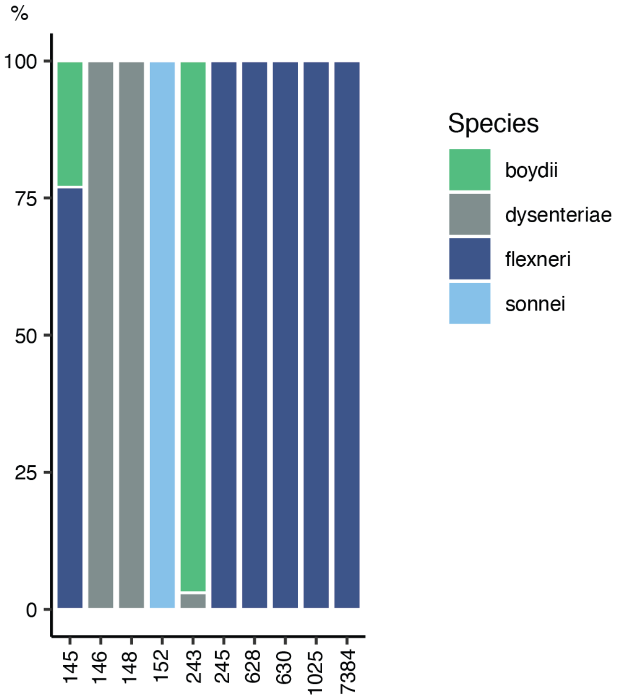

Supplementary Fig 11. Cellulose disruption mutations in *Shigella* spp. *Shigella flexneri* ST245, ST628, ST630, ST1025 and ST7384 possess a nonsense mutation in BcsA, *Shigella sonnei* ST152 contain nonsense mutations in both BcsA and BcsC, and *Shigella dysenteriae* ST146 and ST148 contain nonsense mutations in BcsC, BcsE and BcsG. Source data are provided as a Source Data file.

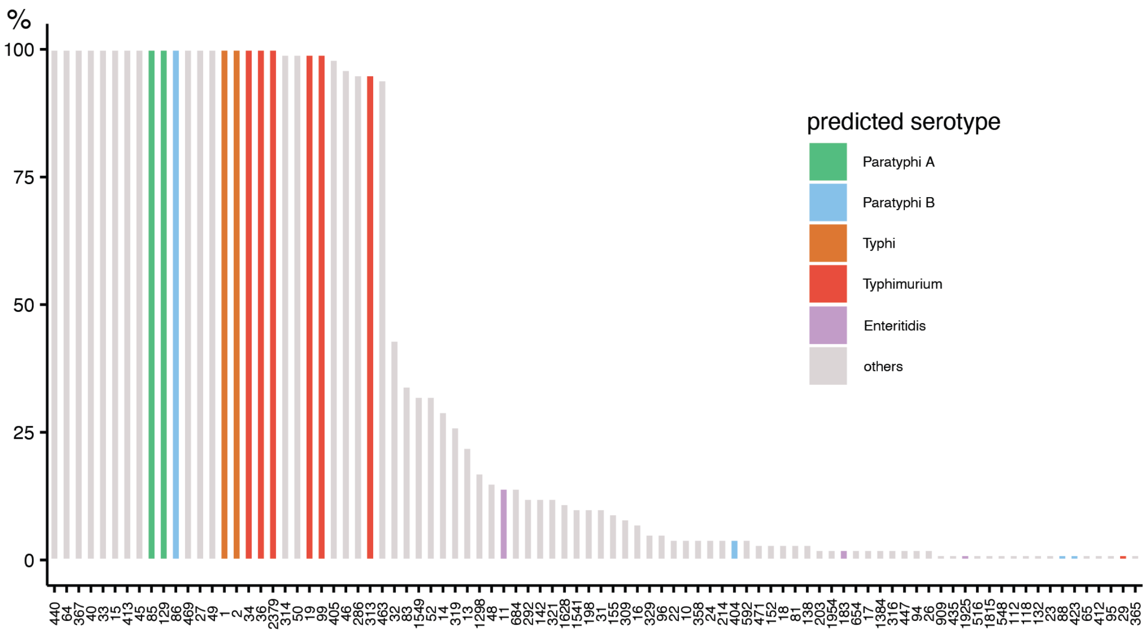

Supplementary Fig 12. Cellulose disruption mutations in *Salmonella* spp. All *S.* Typhimurium STs examined (except ST29) contain disruption mutations in the BcsG pEtN transferase, including 88% (88/100) of the African-endemic invasive nontyphoid bacteremia ST313 clone. In human-restricted *S. enterica* serovars causing typhoid fever (*S.* Typhi) and paratyphoid fever (*S.* Paratyphi A, B and C), loss-of-function mutations in cellulose genes are present in most STs, including a nonsense mutation in the *bcsC* gene in the most abundant *S.* Typhi ST1 and ST2 lineages. Similarly, all *S.* Paratyphi A strains examined from ST85 and ST129 contain a loss-of-function mutation in the *bcsA* gene, while all *S.* Paratyphi B *sensu stricto* strains examined from ST86 contain a nonsense mutation in the *bcsC* gene. In contrast, cellulose disruption mutations were rare in strains belonging to *S.* Paratyphi B *Java*, the other biotype of the *S.* Paratyphi B complex that causes non-invasive gastroenteritis. Source data are provided as a Source Data file.
