# Supplementary material for: A convergent evolutionary pathway attenuating cellulose production drives enhanced virulence of some bacteria
Source: Nat Commun. 2024 Feb 21;15:1441. doi: 10.1038/s41467-024-45176-4 (PMC10881479; doi:10.1038/s41467-024-45176-4)
Supplement: Supplementary file 3 — Description of Additional Supplementary Files [file 41467_2024_45176_MOESM3_ESM.pdf]

Description of Additional Supplementary Files

File Name: Supplementary Data 1
Description: ST95 genomes used in the study

File Name: Supplementary Data 2
Description: Gene content of genomic islands (GIs) in MS7163 and UTI89

File Name: Supplementary Data 3
Description: Prevalence of cellulose disrupted mutations in most common *E. coli* STs

File Name: Supplementary Data 4
Description: Prevalence of cellulose disrupted mutations in most common *Shigella* spp. STs

File Name: Supplementary Data 5
Description: Prevalence of cellulose disrupted mutations in most common *Salmonella* spp. STs

File Name: Supplementary Data 6
Description: Strains and primers used in the study

File Name: Supplementary Data 7
Description: Accession numbers of *E. coli, Shigella and Salmonella* used in the study
